# A case report of Good syndrome: Diarrhea and dyspnea

**DOI:** 10.1097/MD.0000000000049404

**Published:** 2026-07-17

**Authors:** Qingqing Sun, Jinfa Wang, Pingping Hu, Qi Lin

**Affiliations:** a Department of Gastroenterology, The Affiliated People’s Hospital of Ningbo University, Ningbo, China.

**Keywords:** diarrhea, Good syndrome, hypogammaglobulinemia, opportunistic infection, thymoma

## Abstract

**Rationale:**

Good syndrome (GS) is a rare acquired immunodeficiency disorder characterized by thymoma, hypogammaglobulinemia, and peripheral B-cell lymphopenia. Because of its rarity and complexity, the diagnosis and treatment of this condition remain inadequately reported in clinical practice. Therefore, we report a typical case of GS in a patient who developed chronic diarrhea and recurrent pneumonia several years after thymectomy, with the aim of improving the understanding and early recognition of this disease.

**Patient concerns:**

A 55-year-old Chinese woman presented with chronic diarrhea and recurrent severe pneumonia, which developed 3 and 6 years, respectively, after thymectomy for a mixed-type (B2/B3) thymoma.

**Diagnoses:**

Laboratory examination results revealed severe hypogammaglobulinemia (immunoglobulin G = 3.9 g/L), peripheral blood B-cell lymphopenia (B lymphocytes 12 cells/μL), and cellular immune dysfunction (CD4+/CD8+ ratio 0.31, CD(16 + 56): 0.23%, CD19: 3.31%). The diagnosis was confirmed based on the combination of clinical manifestations, laboratory findings, and imaging examinations. GS was diagnosed based on the triad of thymoma history, humoral and cellular immunodeficiency, and recurrent opportunistic infections.

**Interventions:**

The patient received long-term intravenous immunoglobulin (IVIG) replacement therapy, prophylactic antibiotics, and aggressive management of acute infections.

**Outcomes:**

Despite regular IVIG therapy, the patient’s condition progressively deteriorated over 8 years, and the patient ultimately died at home, likely due to respiratory failure secondary to severe pulmonary infection (the family declined an autopsy).

**Lessons:**

This case highlights that GS can manifest several years after thymectomy, necessitating sustained clinical vigilance. This case also highlights the limitations of conventional IVIG therapy and underscores the need to enhance clinicians’ awareness of GS.

## 1. Introduction

Good syndrome (GS), first described by Robert Good in 1955, is a rare acquired immunodeficiency syndrome associated with thymoma.^[1]^ Its classic triad includes thymoma, hypogammaglobulinemia, and peripheral B-cell lymphopenia.^[2]^ The pathogenesis remains unclear, and clinical manifestations typically involve recurrent and refractory opportunistic infections of the respiratory and gastrointestinal tracts. GS predominantly affects adults aged 40 to 60 years.^[3]^ Because of nonspecific symptoms, diagnosis is often delayed, and immunodeficiency may persist even after thymectomy.^[4]^ Current management relies on intravenous immunoglobulin (IVIG) replacement and antimicrobial therapy, yet the prognosis remains poor.^[5]^

Recent advances in immunology have identified multiple signaling pathways and regulatory mechanisms that may inform the understanding of GS. In diffuse large B-cell lymphoma, T-cell exhaustion and immune dysregulation within the tumor microenvironment are central to disease progression.^[6]^ The src homology 2 domain‑containing protein tyrosine phosphatase-1 phosphatase critically regulates CD40-induced mitogen‑activated protein kinase signaling, modulating the balance between pro-inflammatory and anti-inflammatory immune responses interleukin‑11 has been shown to induce encephalitogenic Th17 cells involved in chronic tissue inflammation, and to activate the NLRP3 inflammasome in monocytes, driving IL-1β secretion and inflammatory cell migration.^[7–9]^ Programmed death-1 overexpression on regulatory T-cells impairs IL-10 production and promotes T-cell anergy in chronic infections such as leprosy.^[10]^ Beyond classical immune signaling, protein conformation and stability influence host-pathogen interactions, as demonstrated by pH-dependent conformational changes in the dengue virus NS2B-NS3 protease.^[11]^ Epigenetic and metabolic regulators, including sirtuins, modulate cellular metabolism, oxidative stress, and immune cell function, with distinct roles in pancreatic and prostate cancers.^[12,13]^ From a therapeutic perspective, chimeric antigen receptor T cell therapy has revolutionized the treatment of hematological malignancies by redirecting T-cell specificity against tumor-associated antigens.^[14]^

Despite these mechanistic insights, GS remains under-recognized, and its optimal management is undefined. Herein, we report a typical case of GS with delayed onset of chronic diarrhea and recurrent pneumonia years after thymectomy, aiming to improve early recognition and to highlight potential therapeutic directions, including fecal microbiota transplantation (FMT) as suggested in recent literature, although this patient did not receive such treatment.^[15]^

## 2. Case presentation

A 55-year-old nonsmoking Chinese woman was admitted in May 2010 with a 6-month history of recurrent cough with scant white sputum. Her medical history was unremarkable. Physical examination showed no abnormalities. She was 162 cm tall and weighed 66 kg. Chest computed tomography (CT) revealed an anterior superior mediastinal mass.

She underwent a video-assisted thoracoscopic resection, which was converted to open surgery because of significant intraoperative hemorrhage (approximately 2500 mL). Pathological examination confirmed a mixed-type (B2/B3) thymoma. Postoperative cough resolved, and she received adjuvant radiotherapy. One week later, she developed a left subclavian vein thrombosis and was treated with warfarin (2.5 mg daily) until June 2012.

**Figure 1.**
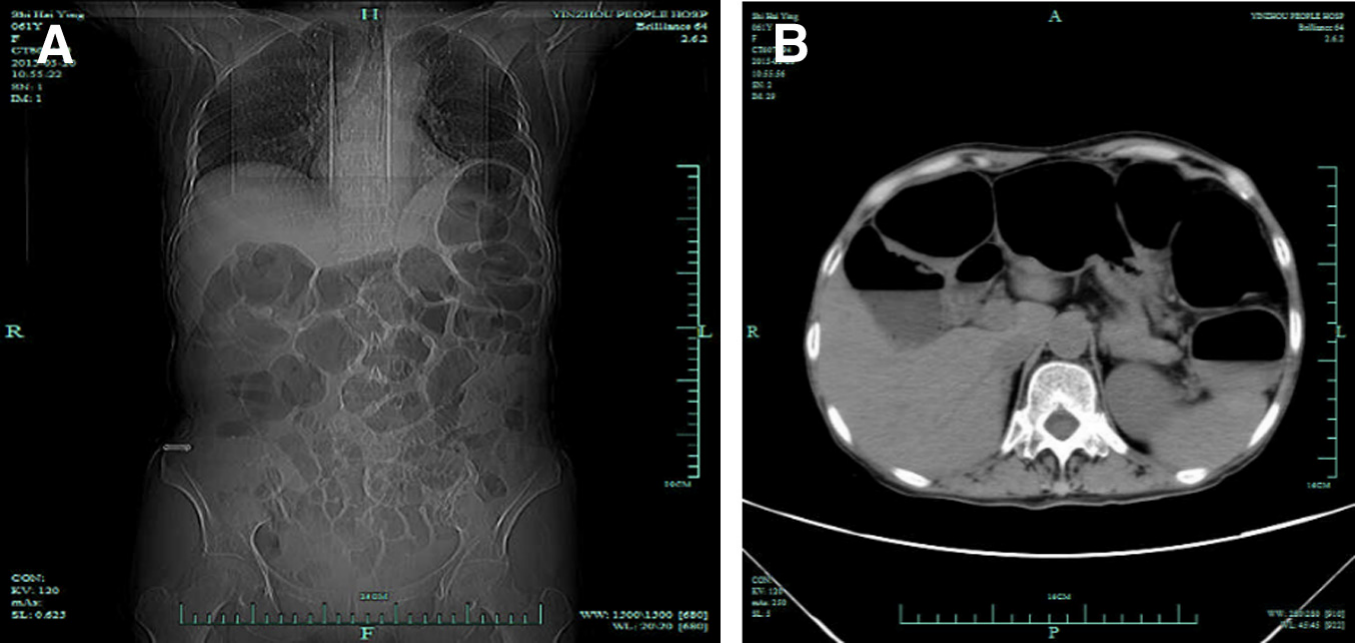
Plain radiography and abdominal CT image of the patient on January 4, 2014. Plain radiography shows marked intestinal fluid accumulation and pneumatosis intestinalis (A). Abdominal CT image reveals multiple air-fluid levels. (B) These findings indicate severe intestinal dysmotility and mucosal barrier disruption, consistent with chronic enteropathy secondary to Good syndrome. CT = computed tomography.

**Figure 2.**
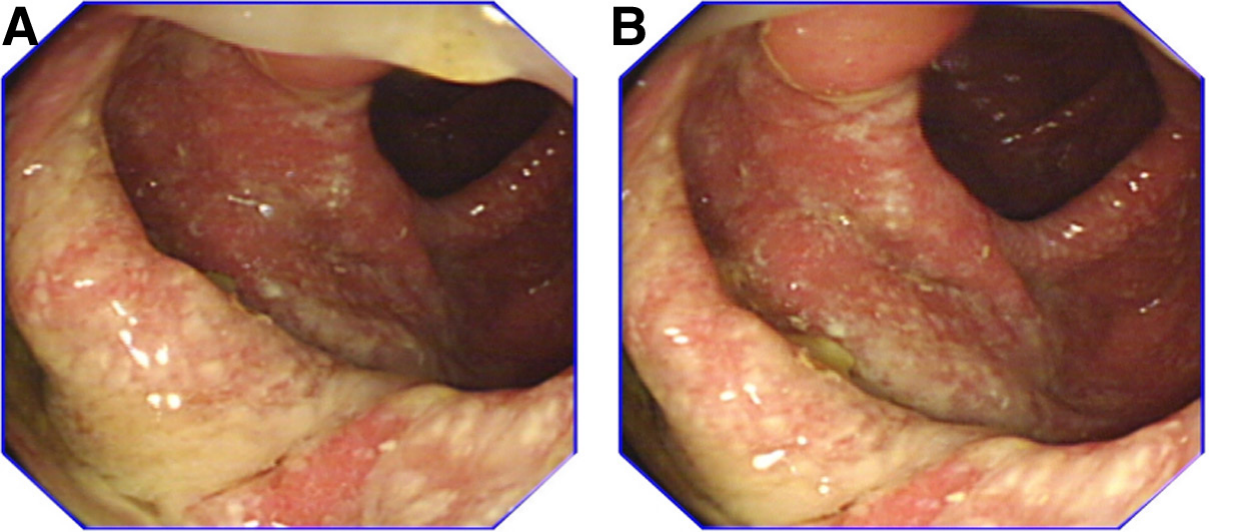
Colonoscopy performed on April 25, 2013. Endoscopic view shows inflammatory changes in the ascending colon, including mucosal erythema, edema, and erosions (A, B). Histopathological examination of the biopsy specimen (hematoxylin and eosin staining) confirms chronic inflammatory cell infiltration in the colonic mucosa and submucosa, with cryptitis and focal mucosal erosion. These features suggest an immune-mediated gastrointestinal involvement in Good syndrome.

**Figure 3.**
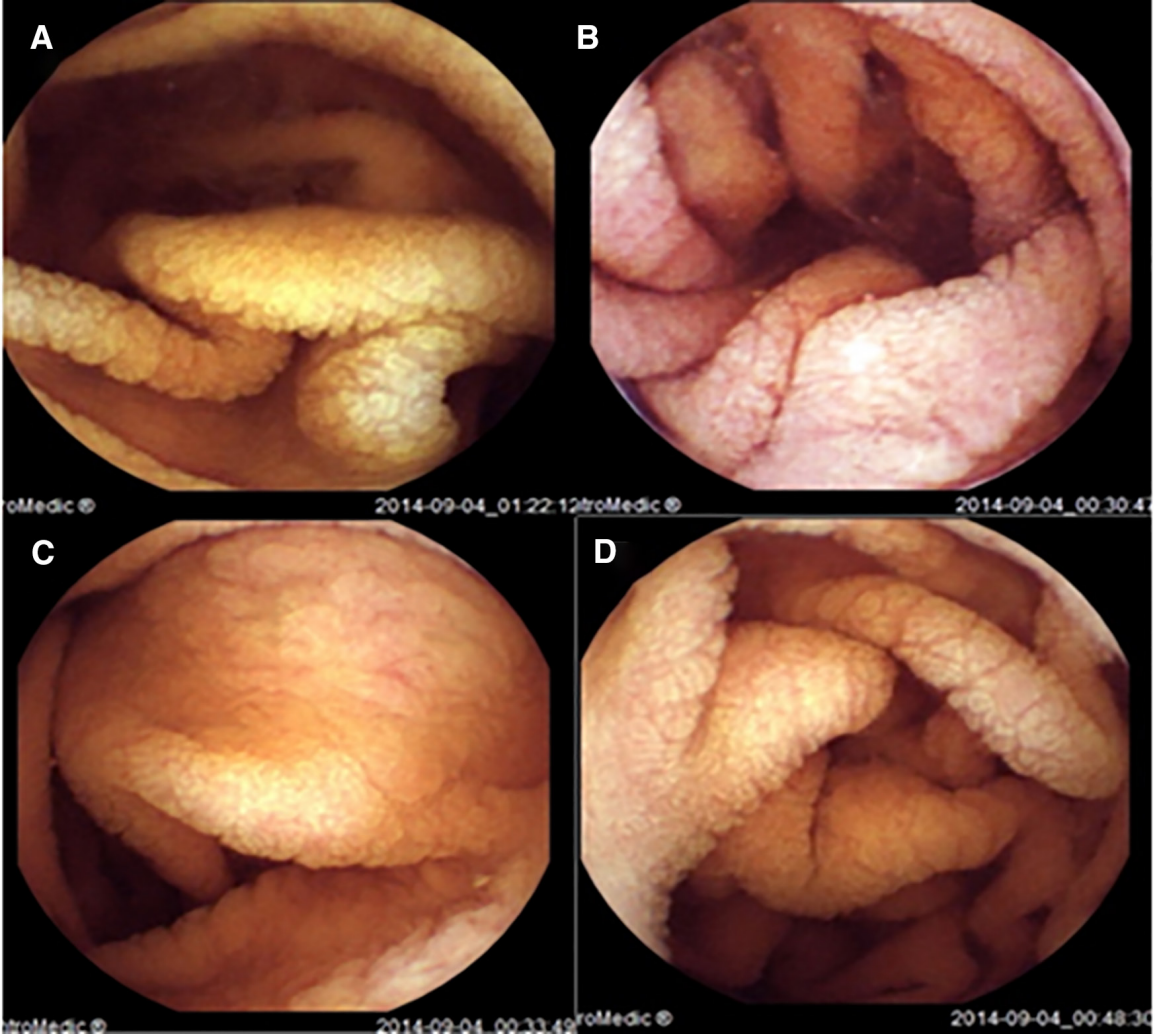
Capsule endoscopy image obtained on September 4, 2014. The image demonstrates diffuse inflammatory alterations of the small intestinal mucosa, characterized by shortened and atrophic villi (A–D). These changes are indicative of chronic malabsorption and enteropathy associated with immunodeficiency in Good syndrome.

In 2013, 3 years after thymectomy, the patient began experiencing chronic diarrhea (2–3 loose stools per day) with a weight loss of 15 kg over 3 years. On December 31, 2013, she was hospitalized because of worsening diarrhea. Laboratory findings revealed a red blood cell count of 3.26 × 10^12^/L (reference range: 3.80–5.10 × 10^12^/L), hemoglobin 118 g/L (reference range: 115–150 g/L), white blood cell count 16.4 × 10^9^/L (reference range: 3.5–9.5 × 10^9^/L), neutrophil percentage 82.4% (reference range: 40%–75%), absolute neutrophil count 38.54 × 10^9^/L (reference range: 1.80–6.30 × 10^9^/L), platelet count 156 × 10^9^/L (reference range: 125–350 × 10^9^/L), lymphocyte count 0.4 × 10^9^/L (reference range: 1.1–3.2 × 10^9^/L), lymphocyte percentage 17.3% (reference range: 20.0%–50.0%), and C-reactive protein 86 mg/L (reference range: 0–8 mg/L). Cellular immune function assessment showed an NK cell count of 50 cells/μL (reference range: 150–1100 cells/μL), CD(16 + 56) 0.23% (reference range: 5.0%–30.0%), T lymphocytes 1155 cells/μL (reference range: 955–2860 cells/μL), CD3 76.81% (reference range: 59.0%–84.0%), B lymphocytes 12 cells/μL (reference range: 90–560 cells/μL), and CD19 3.31% (reference range: 6.0%–22.0%). Severe hypogammaglobulinemia was confirmed, with immunoglobulin G 3.9 g/L (reference range: 7.23–16.85 g/L), immunoglobulin A 0.29 g/L (reference range: 0.69–3.80 g/L), and immunoglobulin M 0.21 g/L (reference range: 0.63–2.77 g/L). Abdominal CT showed intestinal fluid and air accumulation with multiple air-fluid levels (Fig. 1). A previous colonoscopy (April 25, 2013) revealed inflammatory changes in the ascending colon (Fig. 2); histopathological examination of the biopsy specimen from the ascending colon showed chronic inflammatory cell infiltration in the colonic mucosa and submucosa, with cryptitis and focal mucosal erosion. Subsequent capsule endoscopy (September 4, 2014) confirmed enteritis (Fig. 3). Between 2014 and 2016, the patient was hospitalized 13 times for refractory diarrhea. IVIG therapy (2.5 g daily for 3 consecutive days per month) was initiated, providing transient benefit over 5 months before being discontinued because of perceived inefficacy and cost concerns.

**Figure 4.**
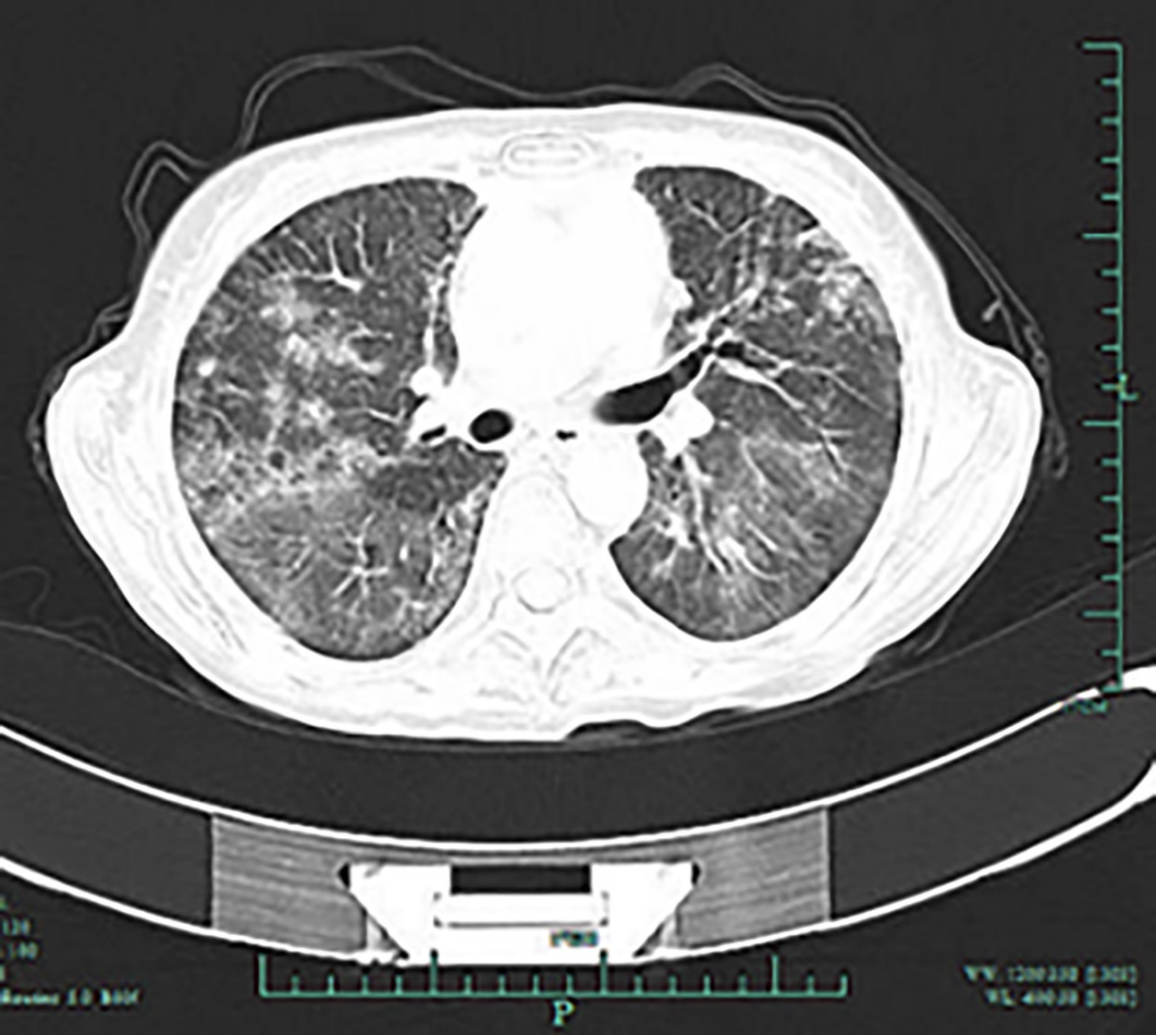
Chest CT image acquired on September 15, 2016. The scan reveals bilateral pulmonary infiltrates, predominantly involving the lower lobes, consistent with recurrent aspiration pneumonia or opportunistic infection. Microbiological workup later identified *Pseudomonas aeruginosa* as the causative pathogen. This finding reflects the profound susceptibility to respiratory infections in patients with Good syndrome. CT = computed tomography.

On September 15, 2016, she was readmitted with cough, productive sputum, and fever (T: 39.5°C). Physical examination revealed extensive moist rales in both lungs. Chest CT revealed bilateral pulmonary infiltrates (Fig. 4). She was treated with broad-spectrum antibiotics and antifungals, with partial response. Over the following 16 months, she experienced multiple episodes of recurrent pneumonia requiring repeated hospitalizations. Pseudomonas aeruginosa was identified microbiologically. Despite aggressive antimicrobial therapy, her respiratory status continued to decline.

**Figure 5.**
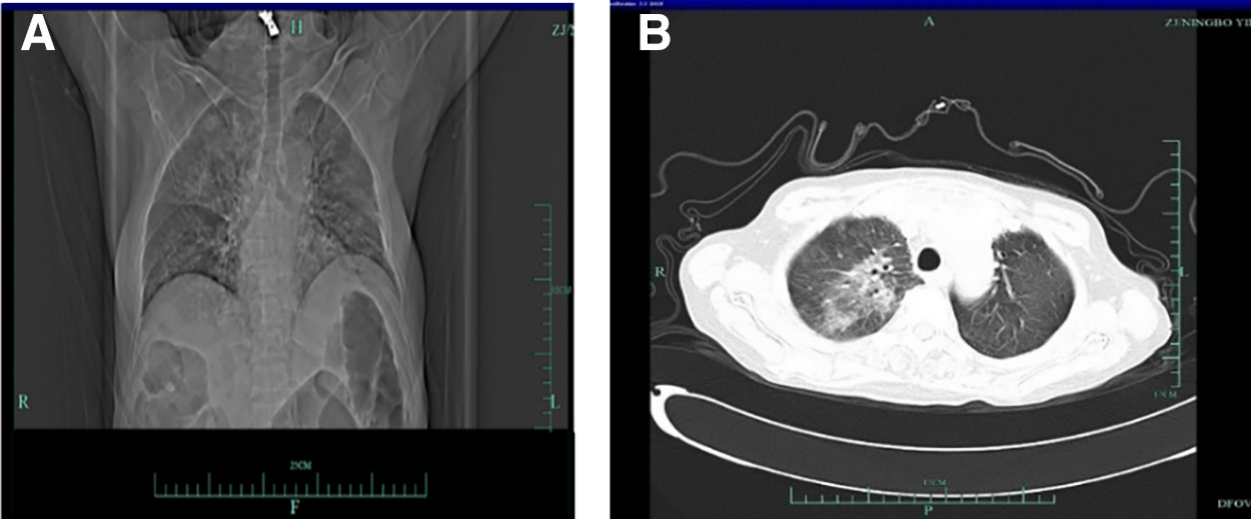
Chest CT image obtained on January 27, 2018, during the patient’s final admission. Compared with previous studies, this scan shows progressive worsening of bilateral pulmonary infectious infiltrates with interstitial changes and a newly developed bulla in the left lower lobe (indicated by the red arrow in the figure) (A, B). The anterior mediastinal region exhibits residual soft tissue opacity. These findings represent end-stage pulmonary damage resulting from recurrent infections in the setting of severe combined immunodeficiency. CT = computed tomography.

During her final admission (January 27, 2018), she presented with cough and progressive dyspnea. Chest CT showed worsened interstitial and alveolar infiltrates (Fig. 5). She developed acute hypoxemic respiratory failure (SpO_2_ 69% on oxygen) and was transferred to the intensive care unit. Immunological workup confirmed persistent humoral and cellular immunodeficiency: immunoglobulin G 4.19 g/L (reference range:7.23–16.85 g/L), CD4 + 16.52% (reference range: 28%–60%), CD8 + 53.23% (reference range: 11%–38%), CD4+/CD8+ ratio 0.31 (reference range: 0.7–2.50). Despite maximal supportive care (meropenem 1.0 g intravenously every 8 hours, linezolid 0.6 g intravenously every 12 hours, and caspofungin 50 mg once daily intravenously, with a 70 mg loading dose), her condition deteriorated. The family requested discharge, and she died at home the next day, likely because of respiratory failure.

**Figure 6.**
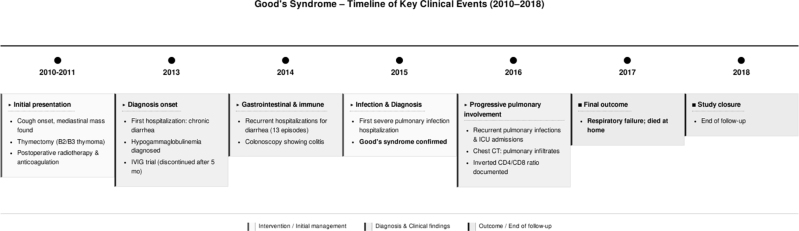
Timeline summarizing the patient’s 8-year clinical course from thymoma resection to death. Key diagnostic milestones, including the identification of hypogammaglobulinemia (2013) and the definitive diagnosis of Good syndrome (2017), are highlighted. Major therapeutic interventions, such as intravenous immunoglobulin (IVIG) therapy and antimicrobial treatments, are also indicated. The timeline clearly illustrates the prolonged diagnostic delay between the onset of chronic diarrhea (2013) and the final diagnosis (2017). CT = computed tomography, ICU = intensive care unit, IVIG = intravenous immunoglobulin.

Figure 6 provides a chronological overview of the patient’s 8-year clinical course, highlighting the key diagnostic milestones and therapeutic interventions, as well as the prolonged delay between symptom onset and definitive diagnosis.

## 3. Discussion

This case report describes a patient with GS who developed chronic intractable diarrhea and recurrent severe pneumonia 3 and 6 years after thymectomy, respectively, with a diagnostic delay of several years. Despite standard IVIG replacement therapy and aggressive antimicrobial treatment, the patient’s condition progressively deteriorated, ultimately leading to death from respiratory failure secondary to severe pulmonary infection.

GS is an exceedingly rare disorder characterized by thymoma, hypogammaglobulinemia, and peripheral B-cell lymphopenia,^[16]^ with a 5-year survival rate of 70% and a 10-year survival rate of 33%.^[17]^ Fewer than 400 cases have been reported globally as of 2023,^[18]^ and the disease shows a worldwide sporadic distribution, with Europe and Asia accounting for 46.9% and 22.8% of cases, respectively.^[19]^ Diagnosis is often delayed because of its heterogeneous presentation, as illustrated by the 3-year diagnostic process in our patient.

The clinical manifestations of GS are highly variable, ranging from thymoma-related symptoms to recurrent infections resulting from combined immunodeficiency.^[3]^ Unlike common variable immunodeficiency, GS typically presents in adults aged 40 to 60 years with recurrent enteritis and pneumonia. Chronic diarrhea occurs in approximately 36% of patients and is often associated with malabsorption because of mucosal pathology or bacterial overgrowth.^[20]^ Fatal opportunistic infections are common in patients with a significant T-cell deficiency.^[21]^ Although most patients present with infections before thymoma diagnosis, our case demonstrated a rare delayed onset, with recurrent diarrhea and respiratory infections emerging 3 and 6 years after thymectomy, respectively, accompanied by progressive weight loss, representing a weight loss of 25 kg over 8 years.

GS lacks a specific biomarker and is diagnosed based on the characteristic triad.^[2]^ Diagnostic challenges arise from its phenotypic heterogeneity and the absence of definitive criteria, often leading to delays or misdiagnosis.^[18]^ Immunological evaluation should be considered in patients with thymoma who present with chronic diarrhea, cytopenia, or recurrent infections,^[22]^ and GS should be included in the differential diagnosis of those with unexplained opportunistic infections.^[23]^

Treatment remains suboptimal, relying on IVIG replacement and antimicrobial therapy.^[18]^ Despite IVIG therapy, our patient showed limited improvement and ultimately died at home, likely because of respiratory failure secondary to recurrent pulmonary infections (the family declined an autopsy to determine the exact cause of death). Outcomes are influenced more by infection severity and associated autoimmune abnormalities than by the thymoma itself.^[24]^ Recently, FMT has emerged as a potential therapeutic strategy. Studies suggest that FMT may restore gut-immune axis function and improve symptoms in refractory cases,^[25]^ offering new hope for GS management.

GS remains incurable even after diagnosis, largely because of profound T-cell mediated immune dysfunction that underlies opportunistic infections.^[26]^ Standard management includes thymectomy followed by IVIG replacement and antimicrobial therapy.^[19]^ Our patient received IVIG (2.5 g × 6 vials, 3 days/month) postdiagnosis; however, after 5 cycles with minimal perceived benefit, she discontinued treatment. She subsequently developed recurrent pneumonia, required repeated intensive care unit admissions, and died shortly after discharge. Clinical outcomes depend more on infection severity and associated hematologic or autoimmune abnormalities than on the thymoma itself, explaining the disease progression despite early thymectomy.^[27]^

A 2019 report suggested a potential role for FMT in GS, documenting symptom improvement without adverse effects such as nausea or vomiting in a 73-year-old patient.^[15]^ FMT has gained increasing attention for its ability to remodel the gut microbiota. Initially effective against recurrent *Clostridium difficile* infection,^[28]^ FMT has since been explored in conditions including metabolic syndrome, diabetes, cancer, and inflammatory bowel diseases.^[29,30]^ The methodology has been refined into washed microbiota transplantation, enhancing standardization and safety.^[31]^ Although FMT was not performed in this patient, emerging evidence suggests it may represent a potential therapeutic option for refractory GS, warranting further clinical investigation.

Intestinal dysbiosis not only mirrors disease progression but also represents a therapeutic target. Restoring gut microbiota can ameliorate clinical manifestations in certain immunodeficiencies.^[22]^ The gut microbiome and host immune system maintain bidirectional communication,^[32]^ with gut microbiota influencing immune cell differentiation and function via metabolites and microbial-associated molecular patterns, thereby modulating regulatory T cells/T helper 17 cells balance and immunoglobulin A production.^[33]^ FMT and specific probiotics may repair intestinal barrier function, regulate immune responses, and potentially reset systemic immunity.^[34]^

In GS, the first reported application of FMT observed no new infections under standardized protocols, supporting its safety.^[15]^ Benefits may derive from global microbial remodeling and precise immunomodulation, avoiding excessive inflammation or secondary infections. Thus, for patients unresponsive to conventional IVIG and albumin therapy, FMT offers a promising therapeutic opportunity by restoring the “gut microbiota–immune axis.”

## 4. Limitations

This study has several limitations. As a single-center case report, its findings are derived from an in-depth analysis of a single case and lack the generalizability of large-sample studies. The patient’s 8-year medical history involved multiple healthcare institutions, resulting in some missing longitudinal immunologic data. Most importantly, although FMT is discussed as a potential therapeutic avenue based on the literature, it was not administered to this patient. Therefore, we cannot provide direct clinical evidence regarding the efficacy or safety of FMT for GS, which represents a significant gap.

## 5. Conclusion

This case underscores the diagnostic challenges and potential for prolonged delay in recognizing GS, particularly when symptoms arise years after thymectomy. Our findings highlight several key features of GS: immunodeficiency-related symptoms can manifest years after thymectomy, necessitating sustained clinical vigilance even in patients who initially appear stable; conventional IVIG therapy, while providing transient benefit, was insufficient to control infections driven by profound T-cell deficiency, underscoring the limitations of current immunoreplacement strategies; and the long diagnostic delay observed in this case reflects the nonspecific presentation of GS and the lack of routine immunological screening in post-thymectomy patients. Clinicians should maintain a high index of suspicion for GS in patients with a history of thymoma who present with chronic, refractory infections, prompting immediate immunological evaluation. While IVIG remains a cornerstone of management, its benefits are often limited. FMT, based on gut microbiota and aimed at regulating the gut-immune axis, may offer a new therapeutic direction for treating refractory cases and warrants further exploration through animal experiments and clinical studies.

## Acknowledgments

We sincerely appreciate the patient for her cooperation in information acquisition, treatment, and follow-up.

## Author contributions

**Writing – original draft:** Qingqing Sun.

**Writing – review & editing:** Jinfa Wang, Qi Lin.

**Conceptualization:** Pingping Hu.

**Investigation:** Pingping Hu.
